# XPS Investigation of the Oxidation States of the As-Deposited Ta Films Prepared by Magnetron Sputtering Technology

**DOI:** 10.3390/ma16237405

**Published:** 2023-11-28

**Authors:** Ming Hu, Zhaowang Li, Xiaoming Gao, Dong Jiang, Zhilu Liu, Longbang Guo, Xu Zhao, Jun He, Jiayi Sun, Lijun Weng, Desheng Wang

**Affiliations:** 1 Key Laboratory of Science and Technology on Wear and Protection of Materials, Lanzhou Institute of Chemical Physics, Chinese Academy of Sciences, Lanzhou 730000, China; lizhaowang@licp.cas.cn (Z.L.); gaoxm@licp.cas.cn (X.G.); jiangd@licp.cas.cn (D.J.); zlliu@licp.cas.cn (Z.L.); longbangg@licp.cas.cn (L.G.); zhaoxu@licp.cas.cn (X.Z.); hejun@licp.cas.cn (J.H.); sunjy@licp.cas.cn (J.S.); wenglj@licp.cas.cn (L.W.); 2 Center of Materials Science and Optoelectronics Engineering, University of Chinese Academy of Sciences, Beijing 100049, China

**Keywords:** sputtering Ta film, XPS analysis, oxidation state

## Abstract

Due to their versatile and unique properties, tantalum-based thin films have been extensively studied. However, tantalum is susceptible to oxidation due to its higher chemical activity, which is crucial regardless of whether oxidations of Ta are beneficial or detrimental. Therefore, the oxidation of Ta during material processing, especially without conscious means, should be taken seriously. In this study, pure Ta films were fabricated by magnetron sputtering under set procedure parameters. The effects of base pressure and substrate temperature on the degree of oxidation of Ta films were investigated. The results revealed that the magnitude of the base pressure directly affects the oxidation state of the as-deposited Ta films. When preferably avoiding the oxidation of sputtered Ta films, the base pressure should be controlled below 4.4 × 10^−4^ Pa. The substrate temperature has little effect on the oxidation state of the as-deposited Ta films under a base pressure ranging from about 10^−2^ Pa to 10^−4^. We hope that this study can provide some references for controlling the oxidation states of Ta involved in relevant film preparation.

## 1. Introduction

In recent years, tantalum-based materials have attracted considerable attention due to their high corrosion resistance [1,2,3,4,5,6], unique electrical properties [7,8], and excellent in vitro and in vivo biocompatibility [9,10,11]. These properties endow Ta-based materials with the possibility for extended applications. Due to the limitations arising from high melting points, manufacturing costs, and resource scarcity, Ta-based films may be the most important form for the application of tantalum compared to the bulk material of tantalum. Furthermore, it is possible to achieve some fantastic combinations of properties by preparing Ta-based films on the surface of other substrate materials.

It is well known that Ta has high chemical activity, and it easily bonds with oxygen and carbon atoms to form oxides/carbides. For Ta-based films, a superficial layer formed with a stable oxidized status on the film’s surface is a key factor in their good mechanical properties and corrosion resistance. The oxidation status of tantalumin Ta-based films also has a major impact on the electrical, optical, and photocatalytic performance of related films. For example, D. Cristea et al. [12] prepared Ta oxynitride thin films with various configurations by magnetron sputtering. Structures ranging from tetragonal β-Ta to fcc-Ta (O,N) to tantalum-based (Ta_x_O_y_, Ta_x_N_y_, and/or Ta_x_O_y_N_z_) crystallites in an amorphous matrix have been obtained. The electrical resistivity varies in a very large domain, starting from 5.29 × 10^−4^ Ωcm up to 1.93 × 10^6^ Ωcm. However, on the other hand, the formation of oxide layers is not always desired for applications of Ta-based films. It is reasonable, in some cases, to protect Ta from oxidation.

So far, several techniques used for preparing Ta-based films have been reported, involving magnetron sputtering [13,14], ion beam assisted deposition (IBAD) [15], filtered cathodic vacuum arc deposition (FCVAD) [16], and chemical vapor deposition (CVD) [17]. Either way, the residual oxygen in the deposition chamber may lead to partial oxidation of the as-deposited Ta films to some extent. This oxidation induced by residual gas could be negligible for some Ta films’ applications, while for other applications, it must be strictly controlled. Although the unintentional result of partial oxidation with respect to as-deposited Ta-based films was often reported in the related studies, there have been few reports elaborating on the effects of deposition parameters on the oxidation status of as-deposited Ta films.

In this study, we deposited pure Ta films by magnetron sputtering. The base pressures and temperatures of the substrate were elaborately controlled during the films’ deposition. The crystal structure and phase compositions of the as-grown sputtered Ta films were examined by X-ray diffraction (XRD). The surface and sub-surface chemistry status of the prepared samples was analyzed using X-ray photoelectron spectroscopy (XPS) in detail. Then the effect of depositing parameters on the oxidation state of as-deposited Ta films was studied. The purpose of this study is to provide a basis for regulating process parameters aiming to control the oxidation state of Ta-based films prepared by vacuum vapor deposition.

## 2. Experimental Procedures

### 2.1. Deposition Conditions

A series of pure Ta films with different thicknesses were deposited by the RF magnetron sputtering technique. The vacuum system affiliated with this device is mainly composed of a cylindrical vacuum chamber with a diameter of 500 mm and a height of 300 mm, a mechanical pump, and a molecular pump in series. Si (100) wafers with dimensions of about 1 cm × 1 cm, which have a thin native oxide surface layer, were used as substrates. Before film preparation, all substrates were cleaned ultrasonically in alcohol and acetone for 15 min in turn, then blow-dried with nitrogen and quickly transferred into the deposition chamber.

Firstly, the vacuum chamber was evacuated to the target base pressure using a mechanical and a molecular pump in series. After that, all samples were sputter-etched under a negative bias of 300 V in an Ar plasma with a pressure of 8.0 Pa for 20 min to remove surface contaminants. Then, the target was pre-sputtered for 10 min with the shutter closed to clean the target surface for every deposition batch. Finally, opening the shutter, the Ta films were deposited. Additionally, the substrate temperature was set to room temperature and 150 °C, respectively. The substrates were stationary during film deposition, and the argon used in all processes of sample preparation had a purity of 99.9%. A Ta target of 3 inches in diameter and a purity better than 99.9 wt.% was used. All depositions were carried out for 1 h. The main parameters corresponding to the samples (labeled for T*_x_*) are detailed in Table 1.

### 2.2. Analyses

The crystal structure and phase composition of the as-deposited thin films were analyzed using an X-ray diffraction instrument (XRD; Philips, X’Pert Rro, Philips, Amsterdam, The Netherlands) using Cu Kα incident radiation. Grazing-incidence XRD inspection of the film samples was performed in the 2 theta mode in the range between 20° and 90° in the step of 0.05°. The angle of incidence was fixed at 2°. The excitation voltage and current were set at 45 kV and 40 mA, respectively.

The chemistry and oxidation states of the as-deposited films were investigated using X-ray photoelectron spectroscopy (XPS; PHI5702 Quantera Scaning X-ray Microprobe, Physical Electronics (PHI), Chanhassen, MN, USA) with monochromatic Al Kα radiation as the excitation source at 14.9 keV and 10 mA. All XPS data were calibrated to the adventitious C1S peak present at 284.8 eV as the reference. The pressure during XPS analysis was better than 2.0 × 10^−7^ Pa. The XPS peak fitting was performed using the Thermo Scientific (Waltham, MA, USA) Avantage v5.9678 software, which models the Gauss–Lorenzian contributions after background subtraction (Shirely).

It is noted that, in order to reduce the impact of atmospheric oxygen as much as possible, two measures were taken. Firstly, each sample was transferred immediately from the deposition vacuum chamber to the XPS analysis vacuum chamber after film deposition was completed. Secondly, the surface and sub-surface were analyzed, respectively, to obtain more accurate information regarding the oxidation states of the Ta films. Here, the surface refers to the topmost surface of the as-deposited films. The sub-surface refers to the region ~15 nm beneath the topmost surface of the as-deposited films, which were prepared by an ion-etching step using an Argon gun of 3 kV for each XPS measurement. Additionally, the morphologies of the films were characterized using a field emission scanning electron microscope (FESEM; Hitachi SU8020, Hitachi, Tokyo, Japan).

## 3. Results and Discussion

### 3.1. Structural Analysis

**Figure 1:**
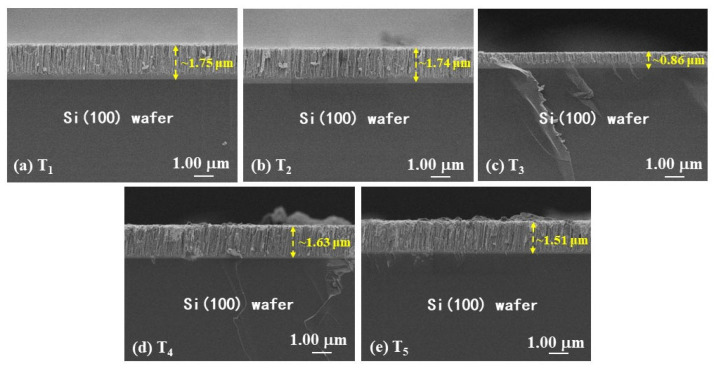
FESEM micrographs of the cross-section of Ta films deposited under different conditions, (**a**) T_1_, (**b**) T_2_, (**c**) T_3_, (**d**) T_4_, and (**e**) T_5_. The thickness of films is revealed by yellow line.

The microstructures of the Ta films deposited under different conditions on Si wafer substrates were characterized using FESEM. The cross-sectional images are shown in Figure 1. Relatively packed columnar structures were observed for all films. The values of thickness for T_1_, T_2_, T_4_, and T_5_ were 1.75 μm, 1.74 μm, 1.63 μm, and 1.51 μm, respectively. It is noted that the thickness of T_3_ has considerably decreased to 0.86 μm. This may be attributed to an increase in the mean free path of the molecular structure due to lower base pressure, which enhances the resputtering effect of the deposited particles.

**Figure 2:**
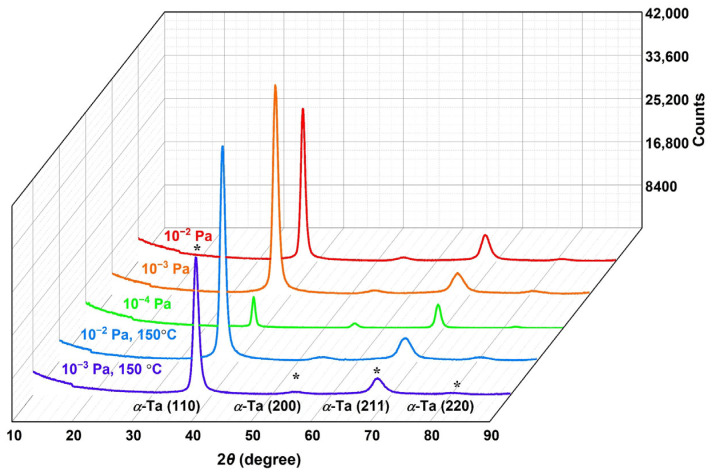
XRD patterns of the Ta films deposited under set parameters. The characteristic lattice planes are marked by asterisk “*”.

Figure 2 shows the X-ray diffractograms of Ta films deposited on silicon substrates with different deposition parameters. The XRD patterns contain distinct (110), (200), (211), and (220) α-Ta reflections, indicating that all the as-deposited films are composed of the α-Ta phase, which is the stable body-centered cubic crystal structure (bcc). The β-phase, which is the tetragonal metastable structure of Ta, was not detected in the as-deposited Ta films in our study. The presented XRD result is consistent with that of the thick Ta-based coating prepared by chemical vapor deposition (CVD) on polished AISI 316L stainless steel [3]. No peaks corresponding to oxide phases were detected from the XRD scan in all cases, indicating that no obvious oxidation occurred for the deposited Ta films when the base pressure ranged from 10^−2^ to 10^−4^ Pa. In addition, the relative intensities of the diffraction peaks for the T_3_ sample obviously decreased compared to the patterns of other samples. The reason for the decrease has not been fully determined, and further research may be required.

### 3.2. XPS Characterization

**Figure 3:**
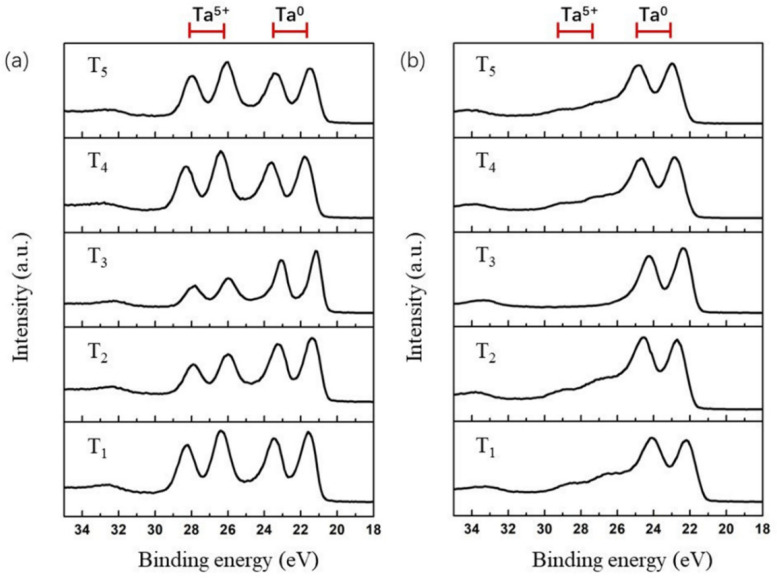
XPS spectra of the Ta 4*f* spectrum acquired from (**a**) the surfaces and (**b**) the sub-surfaces of the Ta films deposited under different parameters.

Figure 3 presents the Ta 4f spectra measured from the surfaces and sub-surfaces of the as-deposited films. Using the known binding energies for the metallic tantalum doublet and the well-reported peak positions of the Ta_2_O_5_ doublet, both pairs of peaks shown in Figure 3a,b can be identified. The contributions (positioned at around 21 eV/23 eV) at the lower binding energy doublet are assigned to the Ta 4*f*_7/2_ and Ta 4*f*_5/2_ peaks of metallic tantalum Ta^0^. The contributions (positioned at around 26 eV/28 eV) at the higher binding energy doublet correspond to the Ta 4*f*_7/2_ and Ta 4*f*_5/2_ peaks of tantalum pentoxide (Ta_2_O_5_) Ta^5+^. The two pairs of Ta^0^ and Ta^5+^ peaks discerned clearly from the Ta 4*f* spectra shown in Figure 3a indicate that both the oxidation and metallic states of tantalum are involved in the surfaces of all the as-deposited Ta films, i.e., partial oxidation of Ta occurred for the surfaces of the Ta films deposited under conditions differing by base pressures and substrate temperatures. But the intensities of the Ta^0^ state relative to those of the Ta^5+^ state increase gradually with decreasing base pressure. Although the effect of exposure to the atmosphere cannot be completely avoided, the variations with respect to the relative intensities of different chemical states of Ta for the T_1_, T_2_, and T_3_ spectra suggest that the content of residual oxygen existing in the films’ deposition chamber would directly have an effect on the surface oxidation degree of the as-deposited Ta films. Interestingly, there were no significant changes in the ratio of Ta^0^ peak intensities to Ta^5+^ peak intensities in the samples deposited under differing substrate temperatures. Therefore, it can be inferred that, with a comparable base pressure level, the substrate temperature would not have a noticeable effect on the oxidation degree of the surface of the as-deposited Ta film.

For the Ta 4*f* spectra measured from the sub-surfaces with a ~15 nm depth, there is a pronounced decrease in Ta^5+^ peak intensities compared to those from the surfaces of corresponding samples. This implies that the oxidation degree of all the samples decreases significantly with increasing depth in all cases. It is important to note that, for the T_3_ sample, the Ta^5+^ doublet located at a binding energy ranging from about 25 eV to 29 eV was no longer observed. This reveals that once the base pressure is lower than 4.4 × 10^−4^ Pa, the residual oxygen in the deposition chamber may have no effect on the deposited Ta films. On the other hand, based on the consistent transfer procedure for all the samples, it could be deduced that the effects of atmospheric exposure are likely to be limited to a depth of ~15 nm.

**Figure 4:**
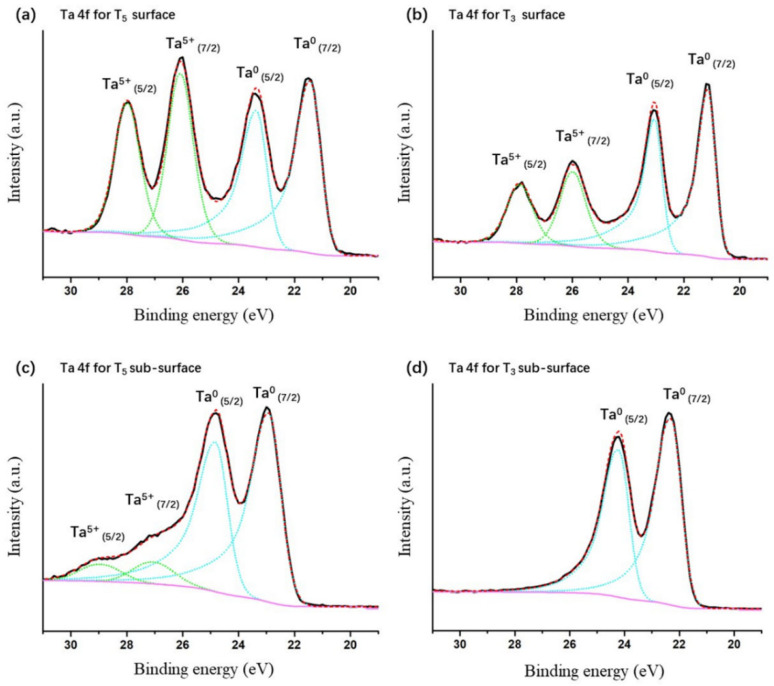
Fitting of the Ta 4*f* spectra for the (**a**) T_5_ surface, (**b**) T_3_ surface, (**c**) T5 sub-surface, and (**d**) T3 sub-surface. The fitting line are colored.

In order to further explore the detailed differences of the oxidation states of the as-deposited Ta films, the deconvolutions of all Ta 4*f* spectra were conducted with a fixed condition where the spin-orbit doublet separation was maintained at 1.9 ± 0.1 eV for all Ta chemical states and the intensity ratios between Ta 4*f*_5/2_ and Ta 4*f*_7/2_ peak at 3/4. Figure 4 shows the typical fitting results (corresponding to the T_3_ and T_5_ samples). The other deconvolution results of the Ta 4*f* spectra relating to the remaining samples are presented in an attached document. The chemical information of all samples, including peak position and peak area, is also given in the attachment (Supplementary Table S1). According to the deconvolution results, the chemical composition of Ta atoms with different valence states for all the studied samples is reported in Table 2.

It is well known that Ta^5+^ (Ta_2_O_5_) [26.5–27.5 eV]/[28.4–29.4 eV] [15] is the most stable oxide state. In addition to Ta^5+^, the other possible sub-oxide reduction states relating to tantalum may also be present, including Ta^4+^ (TaO_2_) [25.4–26.0 eV]/[27.3–27.9 eV], Ta^3+^ (Ta_2_O_3_) [23.0–23.6 eV]/[25.5–24.9 eV], Ta^2+^ (TaO) [24.1–24.5 eV]/[26.0–26.4 eV], and Ta^1+^ (Ta_2_O) [22.4–22.6 eV]/[24.3–24.5 eV] [18], respectively. In order to gain a more accurate peak fitting, all chemical states may be taken into consideration when dealing with Ta 4*f* spectra as a function of the oxidation states of tantalum. However, the lower oxidation states of tantalum can be generated during Ar^+^ bombardment of XPS analysis resulting from the preferential sputtering of O from Ta_2_O_5_ [18]. On the other hand, good fittings of Ta 4*f* envelopes for all the studied samples were obtained when only Ta^5+^ and Ta^0^ doublets were involved. Therefore, in order to avoid overinterpreting the related data, Ta^n+^ corresponding to sub-oxidation states of Ta were excluded in our analyses of the chemical states.

**Figure 5:**
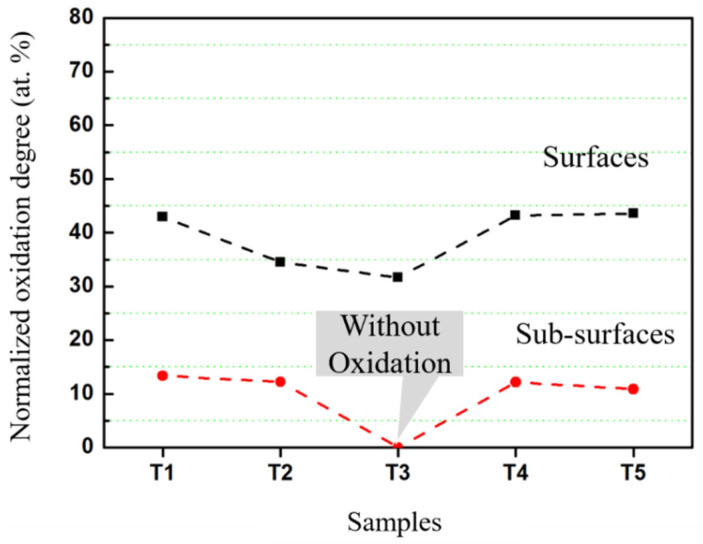
The oxidation degrees of the surfaces and sub-surfaces of the as-deposited Ta films.

According to the peak fitting results of the Ta 4*f* spectra, the chemical states of tantalum from surfaces and sub-surfaces of all the films were analyzed quantitatively. The normalized oxidation degree was defined as the atomic percentage of Ta^5+^ atoms. The oxidation degrees of the surface and sub-surface of each sample are plotted in Figure 5, where the total tantalum atoms are normalized at at. 100%. There are three points to note: (i) for sputtered Ta films deposited under different parameters, the variation trend of oxidation degrees of tantalum on the surface is basically consistent with that of the sub-surface; (ii) a base pressure lower than 4.4 × 10^−4^ Pa should be maintained for sputtered Ta films to be free of oxidation caused by residual oxygen; (iii) the oxidation degrees of the surfaces and sub-surfaces of the samples deposited under heating conditions are comparable to those of the un-heating samples deposited under the same base pressure, respectively.

It is unexpected that the temperature rise of the substrate had no obvious effects on the oxide states of sputtered Ta films. Temperature increases may give rise to the desorption of materials’ surfaces or interfaces. But on the other hand, the chemical reactivity of residual oxygen species should be enhanced by increased temperature. The two effects on the oxidation of deposited films may inhibit each other. Thus, the effects of the substrate temperature on the oxidation states of sputtered Ta films are not definitive and should be further studied in more detail. Additionally, the oxidation degrees of the sub-surfaces of the T_1_, T_2_, T_4_, and T_5_ samples were 13.34, 12.21, 12.14, and 10.86 at. %, respectively. There are no considerable differences between the oxidation degrees of these films. Furthermore, these values are much lower compared to those of the surfaces. This indicates that the overall oxidation levels of the films are not high, which is consistent with the results of the XRD scan showing no reflections corresponding to oxides.

**Figure 6:**
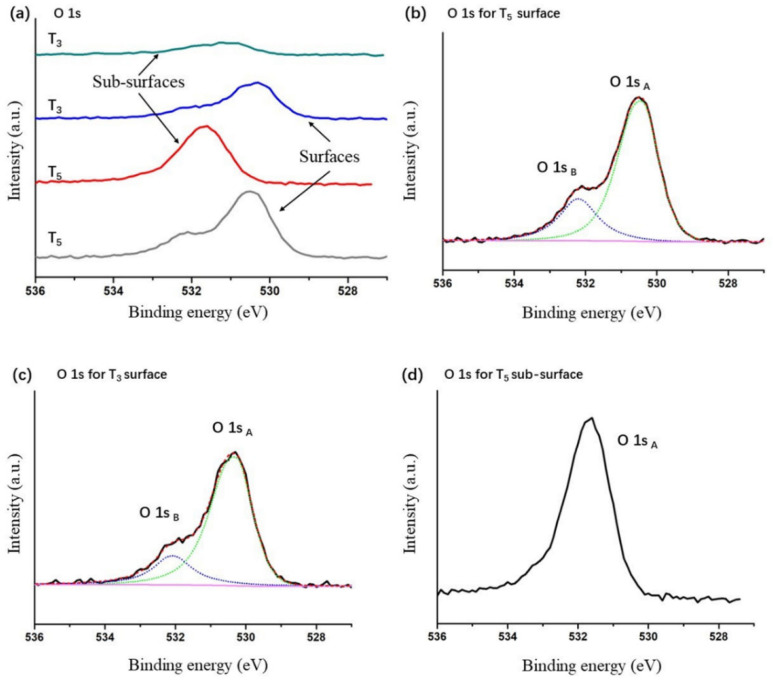
O 1*s* spectra of (**a**) T_3_ and T_5_ with a stack mode, (**b**) T_5_ at the surface, (**c**) T_3_ at the surface, and (**d**) T_5_ at the sub-surface. The fitting lines of O 1*s* spectra are colored.

Figure 6 shows the corresponding O 1s spectra of the T_3_ and T_5_ samples. Comparing the O 1s peaks shown in Figure 6a in stack mode, it was observed that the intensity of the O 1s peak at the sub-surface of T_3_ exhibited a very low intensity. This is consistent with the metallic state (Ta^0^) for the sub-surface of the T_3_ sample verified by XPS analysis, as shown in Figure 4d. The O 1s spectra of the surfaces of T_3_ and T_5_, as shown in Figure 6b,c, differ by the presence of a high-energy shoulder. The peak located at ~532.1 eV is assigned to hydroxylic groups and/or H_2_O adsorbed at the sample surface, and the peak located at ~530.3 eV is assigned to Ta-O bonds in tantalum oxides. The characteristics of the O 1s spectra are very similar to those obtained in previous studies [7]. In addition, for the Ta 4f and O 1s spectra of each sample, one can see that all peaks were shifted slightly towards a high binding energy after Ar^+^ etching was conducted for sub-surface XPS measurements. During the XPS experiments, the negative charge continuously removed from the surface region as a result of a photoelectric effect has to be replenished at a sufficiently high rate to preserve charge neutrality. If this condition is not fulfilled, the surface acquires positive potential, which decreases the kinetic energy of escaping photoelectrons and, in consequence, leads to the apparent shift of all core-level peaks towards higher binding energy [19]. Grzegorz Greczynski and Lars Hultman discussed in detail the relevant issues with using the C 1*s* peak for binding energy calibration [19,20]. As for our experiments, the perfectly accurate determination of the binding energy of Ta and O from XPS spectra is of little significance to this work and is beyond the scope of this work.

## 4. Conclusions

In this study, Ta films with different thicknesses were prepared using RF magnetron sputtering equipment under experimental conditions differing by base pressure and substrate temperature. The crystal structures of the as-deposited Ta films were studied. Especially, the chemical valence states of tantalum in the surface and sub-surface layers of the prepared films were analyzed in detail using XPS. The results show that:

1. The as-deposited Ta films sputtered under different conditions are composed of the α-Ta phase, and none of the films undergo significant oxidation.
2. The residual oxygen concentration in the deposition chamber directly affects the oxidation levels of the Ta films’ surfaces, but the oxidation depth of the film is generally limited to about 15 nm below the surface. As the residual oxygen concentration in the chamber decreases, the oxidation degrees of the surfaces of Ta films also show a significant decrease. When the base pressure of the deposition chamber is lower than 4.4 × 10^−4^ Pa, the Ta films are almost unaffected by the residual oxygen. In addition, it was shown that the substrate temperature has little effect on the oxidation states of the as-deposited Ta films under a base pressure ranging from about 10^−2^ to 10^−4^ Pa.

## Figures and Tables

**Table 1:** Deposition conditions of the sputtered tantalum films.

Samples	Variable Parameters		Fixed Parameters
	Base Vacuum Pressure	Deposition Temperature	
T_1_	4.0 × 10^−2^ Pa	Room temperature	RF power: 100 W; Ar partial pressure: 8.0 × 10^−1^ Pa; Substrate-target distance: 70 mm; Negative bias: 50 V.
T_2_	4.0 × 10^−3^ Pa	Room temperature	
T_3_	4.4 × 10^−4^ Pa	Room temperature	
T_4_	4.0 × 10^−2^ Pa	150 °C	
T_5_	4.0 × 10^−3^ Pa	150 °C	

**Table 2:** The chemical composition (at. %) of Ta atoms with different valence states.

Position	Chemical Composition (at. %)				
	T_1_	T_2_	T_3_	T_4_	T_5_
Surface	57.06/42.94	65.49/34.51	68.36/31.64	56.81/43.19	56.44/43.56
Sub-surface	86.66/13.34	87.79/12.21	100 (Ta^0^)	87.86/12.14	89.14/10.86

## Data Availability

Data are contained within the article and Supplementary Materials.

## Supplementary Materials

The following supporting information can be downloaded at: https://www.mdpi.com/article/10.3390/ma16237405/s1, Figure S1. Decomposition of Ta 4f spectra of (a) T1 surface; (b) T1 sub-surface. Figure S2. Decomposition of Ta 4*f* spectra of (c) T2 surface; (d) T2 sub-surface. Figure S3. Decomposition of Ta 4f spectra of (a) T3 surface; (b) T3 sub-surface. Figure S4. Decomposition of Ta 4*f* spectra of (a) T4 surface; (b) T4 sub-surface. Figure S5. Decomposition of Ta 4f spectra of (a) T5 surface; (b) T5 sub-surface. Table S1. XPS Ta 4*f* binding energies and peak areas employed in the peak fits for the as-deposited Ta films.
