# Supplementary material for: XPS Investigation of the Oxidation States of the As-Deposited Ta Films Prepared by Magnetron Sputtering Technology
Source: Materials (Basel). 2023 Nov 28;16(23):7405. doi: 10.3390/ma16237405 (PMC10707248; doi:10.3390/ma16237405)
Supplement: Supplementary file 1 [file materials-16-07405-s001.zip › materials-2682437-supplementary.pdf]

Supplementary material

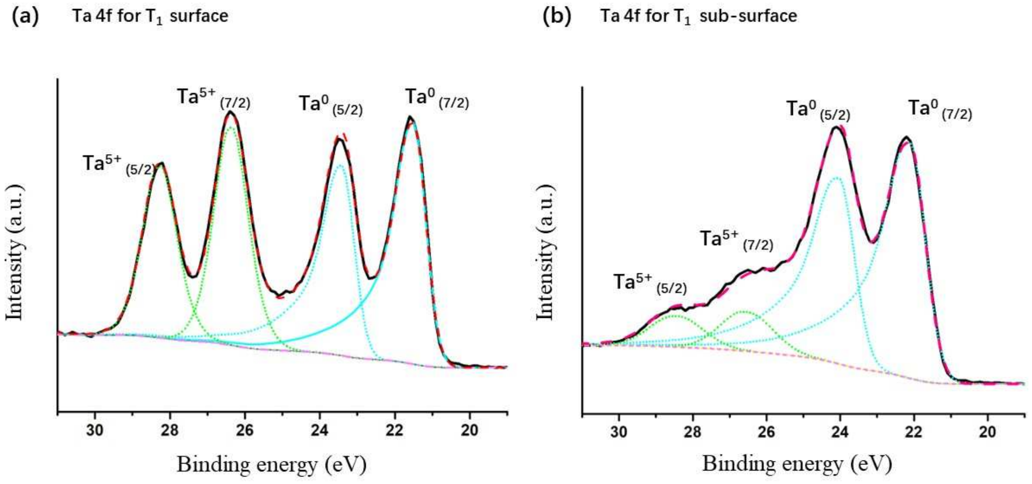

Figure S1. Decomposition of Ta 4f spectra of (**a**) T_1 surface; (**b**) T_1 sub-surface.

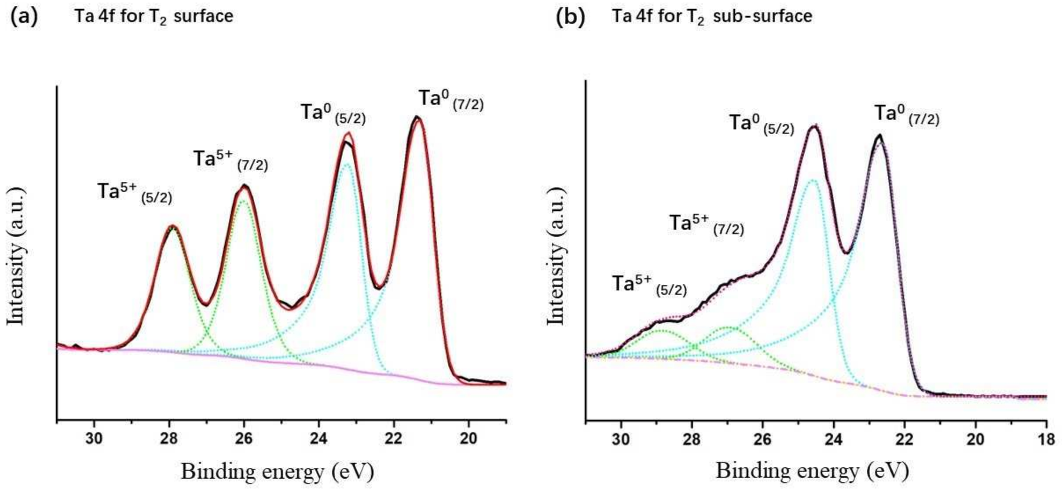

Figure S2. Decomposition of Ta 4f spectra of (**a**) T_2 surface; (**b**) T_2 sub-surface.

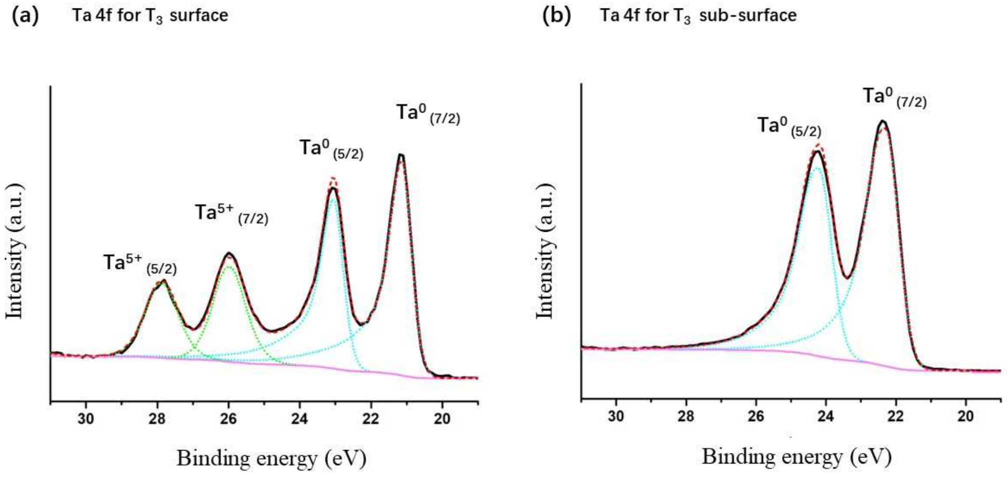

Figure S3. Decomposition of Ta 4f spectra of (**a**) T_3 surface; (b) T_3 sub-surface.

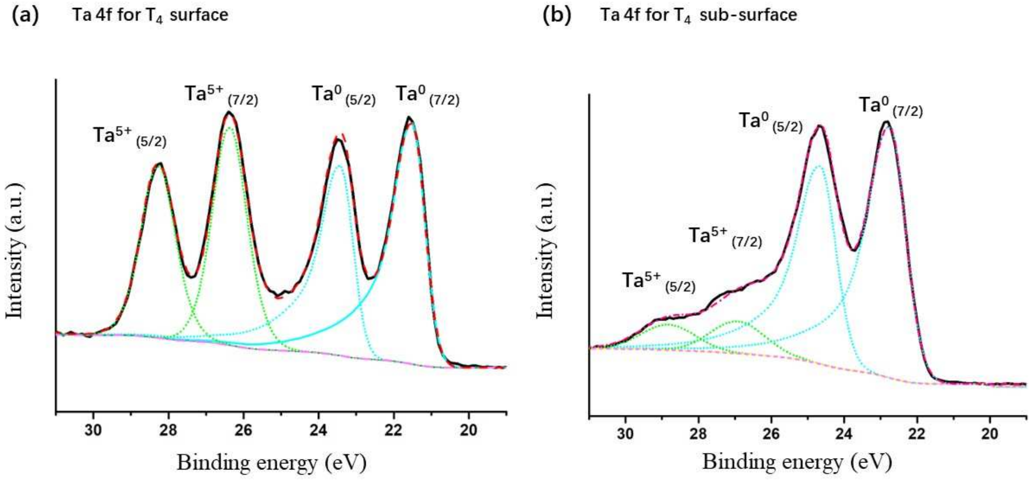

Figure S4. Decomposition of Ta 4f spectra of (a) T_4 surface; (b) T_4 sub-surface.

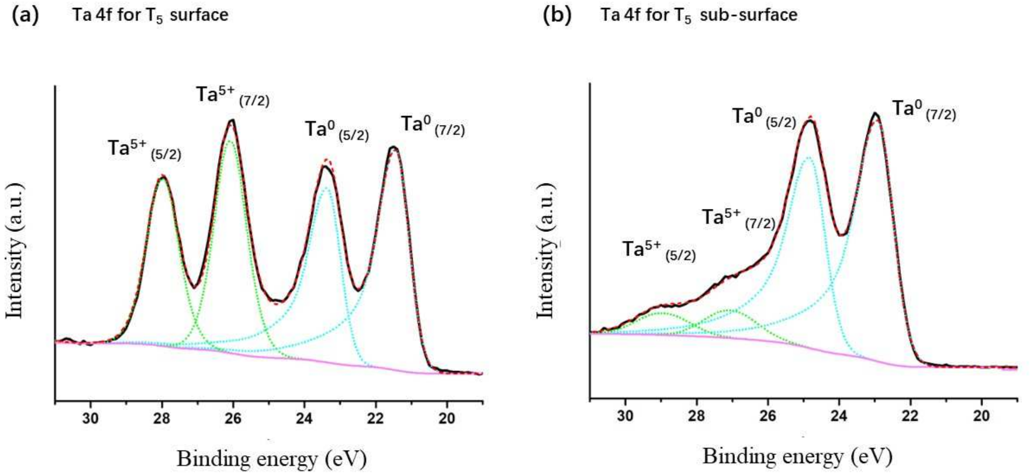

Figure S5. Decomposition of Ta 4f spectra of (a) T_5 surface; (b) T_5 sub-surface.

Table S1. XPS Ta 4f binding energies and peak areas employed in the peak fits for the as-deposited Ta films.

**Chemical States**		Ta^0 [Binding Energy. eV/peak Area CPS. eV]		Ta^{5+} [Binding Energy. eV/peak Area CPS. eV]	
		Ta 4f $_{7/2}$	Ta 4f $_{5/2}$	Ta 4f $_{7/2}$	Ta 4f $_{5/2}$
**Surface**	T_1	21.5 / 17997.39	23.4 / 14203.28	26.4 / 13533.83	28.3 / 10680.69
	T2	21.3 / 23449.37	23.2 / 18505.91	26.0 / 12345.72	27.9 / 9743.06
	T3	21.2 / 12650.74	23.1 / 9983.70	26.0 / 5850.57	27.9 / 4617.19
	T4	21.7 / 16076.98	23.6 / 12687.64	26.4 / 12211.33	28.3 / 9637.01
	T5	21.5 / 14665.35	23.4 / 11573.69	26.1 / 11308.88	28.0 / 8924.81
Sub-surface	T1	22.1 / 41851.99	24.0 / 33021.80	26.5 / 6439.26	28.4 / 5081.77
	T2	22.6 / 55400.32	24.5 / 43721.08	26.9 / 7696.29	28.8 / 6073.80
	T3	22.3 / 32578.73	24.2 / 25654.99	/	/
	T4	22.7 / 36969.62	24.6 / 29175.28	26.9 / 5106.92	28.8 / 4030.31
	T5	22.9 / 36619.56	24.8 / 28899.62	27.0 / 4459.67	28.9 / 3519.51
